# A new prediction model for patient satisfaction after total knee arthroplasty and the roles of different scoring systems: a retrospective cohort study

**DOI:** 10.1186/s13018-021-02469-4

**Published:** 2021-05-20

**Authors:** Jinyu Liu, Yi Yang, Shengcheng Wan, Zhenjun Yao, Ying Zhang, Yueqi Zhang, Peng Shi, Chi Zhang

**Affiliations:** 1 Department of Orthopedic Surgery, Zhongshan Hospital, Fudan University, Shanghai, 200032 China; 2 Children’s Hospital of Fudan University, 399 Wanyuan Road, Shanghai, 201102 China

**Keywords:** Total knee arthroplasty, Patient satisfaction, Score systems, Prediction of satisfaction

## Abstract

**Background:**

Although total knee arthroplasty (TKA) is an efficacious treatment for end-stage osteoarthritis, ~20% of patients are dissatisfied with the results. We determined which factors contribute to patient satisfaction and compared the various scoring systems before and after surgery.

**Methods:**

In this retrospective cohort study, 545 patients were enrolled and evaluated preoperatively and 1 year postoperatively. Patient demographics, as well as scores for the Western Ontario and McMaster Universities Osteoarthritis Index (WOMAC), Short Form (SF)-12, and 1989 Knee Society Clinical Rating System (1989 KSS), were recorded preoperatively and postoperatively. The possible predictors were introduced into a prediction model. Scores for overall satisfaction and the 2011 Knee Society Score (2011 KSS) were also assessed after TKA to identify the accuracy and agreement of the systems.

**Results:**

There were 134 male patients and 411 female patients, with an overall prevalence of satisfaction of 83.7% 1 year after surgery. A history of surgery (*p* < 0.001) and the 1989 KSS and SF-12 were of the utmost importance in the prediction model, whereas the WOMAC score had a vital role postoperatively (change in WOMAC pain score, *p* < 0.001; change in WOMAC physical function score, *p* < 0.001; postoperative WOMAC pain score, *p* = 0.004). C-index of model was 0.898 > 0.70 (95% confidence interval (CI): 0.86-0.94). The Hosmer-Lemeshow test showed a *p* value of 0.586, and the AUC of external cohort was 0.953 (sensitivity=0.87, specificity=0.97). The agreement between the assessment of overall satisfaction and the 2011 KSS satisfaction assessment was general (Kappa=0.437 > 0.4, *p* < 0.001).

**Conclusion:**

A history of surgery, the preoperative 1989 KSS, and the preoperative SF-12 influenced patient satisfaction after primary TKA. We recommend the WOMAC (particularly the pain subscale score) to reflect overall patient satisfaction postoperatively.

## Introduction

Total knee arthroplasty (TKA) is an efficacious treatment to improve the function and quality of life of patients with end-stage osteoarthritis of the knee [1–3]. Despite its widespread use and popularity, several studies have reported that ~20% of patients are dissatisfied with the outcomes of primary TKA [3, 4]. Preoperative expectations, the type of prosthesis, sex, age, and psychological factors have been suggested to be related to this low patient satisfaction [3–8]. However, a “gold standard” questionnaire or tool for the assessment of patient satisfaction is lacking, meaning that there are variable results in terms of preoperative predictors [2, 7].

In recent decades, patient satisfaction and patient-reported outcome measures have become increasingly valued in the assessment of the overall outcome of surgical procedures from the viewpoint of patients rather than that of surgeons [2, 5, 7]. In a recent review, Kahlenberg et al. demonstrated that the most commonly used method for measuring satisfaction is a single question about overall satisfaction that can be answered on an ordinal scale (e.g., “very satisfied,” “somewhat satisfied,” “dissatisfied,” “very dissatisfied”), whereas other scholars have used different Likert scales or multiple questions to assess satisfaction [9]. The different methods of satisfaction reporting and scoring systems lead to difficulty in identifying which patients are truly dissatisfied with the outcomes of primary TKA [3, 8, 9]. Moreover, few scholars have compared the different methods of measuring patient satisfaction or assessed the correlation between the focus of restoration and a postoperative scoring system.

We aimed to assess the prevalence of patient satisfaction after TKA, identify the independent predictors of patient satisfaction preoperatively, and establish a prediction model that could aid in the management of patient satisfaction before and after surgery. Additionally, we aimed to assess the roles of different scales postoperatively, including the association between the focus of questions and various scoring systems. Moreover, we wanted to ascertain the accuracy and agreement between patient satisfaction using the 2011 Knee Society Score (2011 KSS) and overall patient satisfaction.

## Materials and methods

### Ethical approval for the study protocol

The study protocol was approved by the Ethics Committee of Zhongshan Hospital, Fudan University (B2020-234R). All patients provided written informed consent for the use of their data in the present study.

### Inclusion and exclusion criteria

Only patients with primary osteoarthritis were eligible for inclusion in the study. Patients who underwent a second TKA or revision arthroplasties, lacked the ability to complete questionnaires, or had a periprosthetic joint infection during the study period were excluded.

### Study design

This was a retrospective cohort study with a prospectively compiled arthroplasty database in a single institution. Between May 2016 and August 2019, 722 patients undergoing TKA were recruited for our study to establish a predictive model. After the model was built, we used an external cohort, including 101 patients undergoing TKA after August 2019, to assess its performance. Demographic and clinical data were collected based on medical records in the hospital database before surgery.

The scores of various scales were recorded preoperatively using the following established questionnaires: Western Ontario and McMaster Universities Osteoarthritis Index (WOMAC) [10], Short Form (SF)-12 [11], and 1989 Knee Society Clinical Rating System (1989 KSS) [12].

**Fig. 1:**
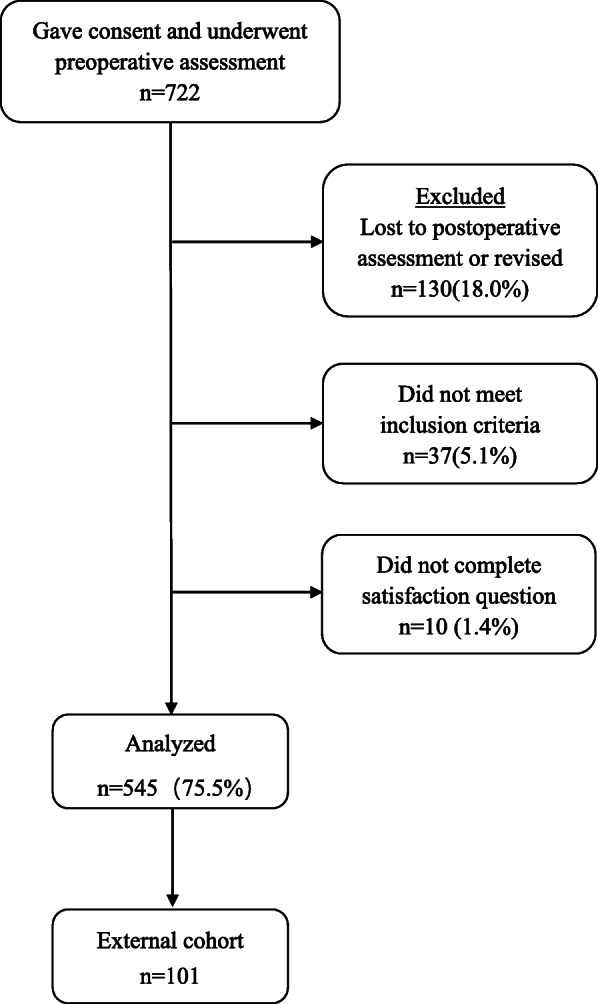
Flowchart illustrating the study cohort

One year after TKA, each patient was asked to complete another questionnaire consisting of the WOMAC, 1989 KSS, SF-12, and 2011 KSS as well as overall satisfaction [13]. Finally, 545 of the 722 patients who underwent primary TKA and had complete form data were included in the study according to the inclusion criteria (Fig. 1).

The data were collected by participating surgeons and their staff in the hospital. Preoperatively, in addition to the answers to various scoring systems, the following data were collected: age, sex, unilateral or bilateral TKA, primary diagnosis, comorbidities, body mass index (BMI), living status (live alone: yes/no), Kellgren–Lawrence grade of osteoarthritis [14], and previous surgery. The type of prosthesis was recorded, and there was no patella resurfacing during TKA.

One year after TKA, we measured patient satisfaction by asking an overall satisfaction assessment question [9] and four satisfaction assessment questions of different major aspects (surgical procedure, functional restoration, pain relief, and fulfillment of expectation). The questions were as follows: (1) “Overall, how satisfied are you with the results of your knee replacement surgery?;” (2) “How satisfied are you with the surgical procedure for your knee replacement surgery?;” (3) “How satisfied are you with the results of your knee replacement surgery for improving your functional abilities (such as standing, walking, and bathing)?;” (4) “How satisfied are you with the results of your knee replacement surgery for relieving your pain?;” and (5) “How satisfied are you with the expectation fulfillment of your knee replacement surgery?.”

The response to each question was recorded using a five-point Likert scale: “very satisfied,” “satisfied,” “neutral,” “dissatisfied,” and “very dissatisfied.” The patients were divided into two groups according to their answer to the question on overall satisfaction. Patients who answered “very dissatisfied,” “dissatisfied,” or “neutral” were assigned to one group, and patients who answered “satisfied” or “very satisfied” were assigned to a second group. This two-category outcome (“satisfied” vs “not satisfied”/“neutral”) was used as the measure of overall satisfaction for all statistical analyses because patient satisfaction or patient dissatisfaction were our primary variables. In addition, the outcomes of the postoperative score and changes in the WOMAC, 1989 KSS, 2011 KSS, and SF-12 scores were also collected and calculated.

The classical 1989 KSS we used included measurements of the knee and functional outcomes by the surgeon. The 1989 KSS scoring system for measuring clinical outcomes has been validated [12]. We also used the new 2011 KSS [13, 15] to assess the outcomes of surgery postoperatively, which has four categories: symptoms, patient satisfaction, patient expectations, and functional activities. In recent years, most researchers have preferred to use the 2011 KSS because it takes patients’ feelings into account, and TKA outcomes are measured from different dimensions [15–18].

The WOMAC [10] consists of three subscales: pain, physical function, and stiffness. The WOMAC comprises 24 questions on a five-point Likert scale (“none,” “mild,” “moderate,” “severe,” and “extreme”). According to recent recommendations, we used the reverse option, from 0 (“worst”) to 100 (“best”) [19–22].

The SF-12 is used frequently to measure well-being. It comprises a physical component summary (PCS) and a mental component summary (MCS). The PCS and MCS range from 0 (“worst level of functioning”) to 100 (“best level of functioning”) according to recommendations [11].

### Statistical analysis

Statistical analyses were performed using SPSS 23.0 (IBM, Armonk, NY, USA) and R 4.0.2 (R Foundation for Statistical Computing, Vienna, Austria). Different variables were compared between the “satisfied” and “dissatisfied” TKA groups. Categorical variables were tested using the chi-square test. Continuous variables were tested using the independent Student’s *t* test or Mann–Whitney *U* test between two groups. Correlation analyses were employed to identify the efficiency of the change in score and postoperative score for reflecting patient satisfaction. Univariate and multivariate logistic regression analyses were performed to identify independent predictors of satisfaction after surgery. A nomogram based on the prediction model and calibration plots were created with the “rms” package. The predictive performance of the nomogram was measured internally by the concordance index (C-index), Hosmer-Lemeshow test and calibration with 1000 bootstrap samples. The discriminatory ability of the nomogram was quantified using Harrell’s concordance index, which ranges from 0.5 to 1.0. Generally, a C-index value greater than 0.70 is considered to represent relatively good discrimination. Additionally, we used the external validation cohort to assess the model externally, and the area under the curve (AUC) of the receiver operating characteristic (ROC) curve was estimated using the “ROCR” package.

To identify the possible predictors of the four aspects of satisfaction (surgical procedure, functional restoration, pain relief, and fulfillment of expectation) preoperatively, we analyzed the preoperative scores and the change in the scores of the relevant subscales (surgical outcomes of the 1989 KSS, 2011 KSS, WOMAC, and SF-12; functional restoration outcome using the function score from the 1989 KSS and the function score from the WOMAC; pain relief outcome using the pain score from the WOMAC; and expectation fulfillment outcome using the expectation score from the 2011 KSS). The 2011 KSS contains an assessment of patient satisfaction as well, so we compared it with overall patient satisfaction by the McNemar and Kappa tests. *P* < 0.05 was considered significant.

## Results

### Patient satisfaction and prediction model

The patient demographics, comorbidities, relevant clinical data and scores of different scales for the study cohort are illustrated in Tables 1 and 2, respectively. There were 134 male patients and 411 female patients, with a mean age of 72.2 years and a mean body mass index (BMI) of 26.1. There was a significant improvement in all scoring systems post-operatively.

**Table 1:** Univariate statistical analysis results of demographic and clinical variables between satisfaction outcome groups (satisfied/very satisfied, dissatisfied/very dissatisfied/neutral)

Variables	Satisfied (*n* = 456)	Dissatisfied (*n* = 89)	Significance (*p*)
Age (mean±SD)	72.02±6.60	73.05±6.70	0.147
BMI (mean±SD)	26.01±3.47	26.21±3.58	0.629
Female	337 (73.9%)	74 (83.1%)	0.064
Unilateral (n, %)	425 (93.2%)	86 (96.6%)	0.221
Comorbidity (*n,* %)	340 (74.6%)	61 (68.5%)	0.239
Live alone (*n,* %)	33 (7.2%)	8 (9.0%)	0.567
K-L grades (mean±SD)	3.76±0.42	3.78±0.43	0.704
Implant type (*n,* %)			0.218
GII	134 (29.4%)	32 (36.0%)	
LEGION	322 (70.6%)	57 (64.0%)	
Previous surgery history (*n,* %)	256 (56.1%)	69 (77.5%)	< 0.001*
Orthopedic surgery history (*n,* %)	129 (28.2%)	30 (33.7%)	0.183
Arthroscopy surgery history (*n,* %)	40 (8.8%)	11 (12.3%)	0.288
Frequency < 2 (*n,* %)	317 (69.5%)	66 (74.2%)	0.228
Frequency < 2 (*n,* %)	405 (88.8%)	82 (92.1%)	0.234

*Statistically significant

**Table 2:** Univariate statistical analysis results of various scoring systems between satisfaction outcome groups (satisfied/very satisfied, dissatisfied/very dissatisfied/neutral)

Variables	Satisfied (*n* = 456)	Dissatisfied (*n* = 89)	Significance (*p*)	Correlation coefficient between variables and overall satisfaction (r)
Preoperative 1989 KSS				
Knee (mean±SD)	48.66±10.85	35.61±12.03	<0.001*	−0.372
Function (mean±SD)	39.21±9.02	21.91±18.66	<0.001*	−0.370
Total (mean±SD)	87.87±15.19	57.52±27.54	<0.001*	−0.432
Postoperative 1989 KSS				
Knee (mean±SD)	85.66±9.87	71.97±18.74	<0.001*	−0.292
Function (mean±SD)	82.11±13.00	48.54±25.30	<0.001*	−0.493
Total (mean±SD)	167.77±16.84	120.51±38.61	<0.001*	−0.504
Change 1989 KSS				
Knee (mean±SD)	37.00±12.84	36.36±16.39	<0.001*	−0.011
Function (mean±SD)	42.89±14.39	26.63±13.65	<0.001*	−0.390
Total (mean±SD)	79.89±19.46	62.99±22.31	<0.001*	−0.283
Preoperative WOMAC				
Pain (mean±SD)	27.99±10.98	25.56±13.21	0.031*	−0.084
Joint (mean±SD)	58.83±23.92	48.17±23.88	<0.001*	−0.166
Function (mean±SD)	36.22±9.07	33.65±9.82	0.016*	−0.106
Total (mean±SD)	41.01±9.96	35.80±10.22	<0.001*	−0.183
Postoperative WOMAC				
Pain (mean±SD)	87.73±10.75	59.38±23.15	<0.001*	−0.470
Joint (mean±SD)	86.27±14.81	75.62±16.69	<0.001*	−0.248
Function (mean±SD)	64.65±9.63	50.63±12.17	0.790	−0.405
Total (mean±SD)	79.55±7.76	61.87±11.36	<0.001*	−0.523
Change WOMAC				
Pain (mean±SD)	59.75±12.74	33.81±22.64	<0.001*	−0.427
Joint (mean±SD)	27.44±20.11	27.44±16.79	0.019*	0.005
Function (mean±SD)	28.43±12.26	16.97±13.70	<0.001*	−0.290
Total (mean±SD)	38.54±9.12	26.08±11.79	0.002*	−0.378
Preoperative SF-12				
PCS (mean±SD)	35.59±7.27	28.42±5.34	0.002*	−0.371
MCS (mean±SD)	32.52±6.70	29.03±5.67	<0.001*	−0.207
Postoperative SF-12				
PCS (mean±SD)	49.71±5.26	39.46±8.69	<0.001*	−0.454
MCS (mean±SD)	43.95±6.43	38.60±6.45	<0.001*	−0.280
Change SF-12				
PCS (mean±SD)	14.12±7.66	11.04±7.22	<0.001*	−0.155
MCS (mean±SD)	11.43±8.50	9.57±8.85	0.062	−0.070
Postoperative 2011 KSS				
Symptom (mean±SD)	15.52±2.33	12.24±2.64	<0.001*	−0.402
Satisfaction (mean±SD)	29.31±6.93	16.48±7.52	<0.001*	−0.495
Expectation (mean±SD)	12.17±2.23	6.96±2.59	<0.001*	−0.551
Function (mean±SD)	67.80±13.02	37.66±15.51	<0.001*	−0.543

*1989 KSS* 1989 Knee Society Clinical Rating System, *WOMAC* Western Ontario and McMaster Universities Osteoarthritis Index, *SF-12* 12-Item Short-Form Health Survey, *PCS* Physical Component Score, *MCS* Mental Component Score, *2011 KSS* 2011 Knee Society Score

*Statistically significant

Overall satisfaction represented the satisfaction outcome overall. A total of 456 patients claimed that they were satisfied or very satisfied, whereas 89 patients were very dissatisfied, dissatisfied, or neutral. There was an obvious difference in postoperative scores and change in scores between the two groups (Table 2). In terms of the different focuses of the assessment of satisfaction, 83.5% of patients were satisfied with the surgical procedure, 81.7% of patients were satisfied with the postoperative functional restoration, 79.6% of patients were satisfied with the pain relief, and 82.0% of patients were satisfied with their fulfillment of expectation.

For the preoperative predictors of overall satisfaction identified by binary logistic regression analysis, dissatisfaction with the overall outcome was more likely in patients who had previously undergone surgery. Further analyses using the chi-square test revealed no significant differences in the prevalence and type of previous surgical procedure (Table 1). Regarding the preoperative scores, patients were more likely to feel dissatisfied if they had lower 1989 KSS or SF-12 scores (Table 3). To establish a prediction model to assess the possibility of dissatisfaction, a nomogram was constructed on the basis of the 1989 KSS and SF-12 and previous surgical history.

**Table 3:** Predictors of overall patient satisfaction after TKA on multivariate analysis

	B (95% CI)	*p* value
Preoperative scores		
1989 KSS knee score	0.048 (1.021, 1.077)	<0.001*
1989 KSS function score	0.066 (1.041, 1.097)	<0.001*
WOMAC pain score	0.016 (0.991, 1.042)	0.220
WOMAC joint score	0.008 (0.996, 1.021)	0.206
WOMAC function score	0.012 (0.979, 1.046)	0.488
PCS score	0.071 (1.022, 1.128)	0.004*
MCS score	0.127 (1.077, 1.196)	<0.001*
Change scores		
1989 KSS knee score	−0.004 (0.973, 1.019)	0.708
1989 KSS function score	0.051 (1.030, 1.076)	<0.001*
WOMAC pain score	0.074 (1.056, 1.099)	<0.001*
WOMAC joint score	−0.007 (0.977, 1.010)	0.423
WOMAC function score	0.050 (1.025, 1.079)	<0.001*
PCS score	0.019 (0.979, 1.061)	0.355
Postoperative scores		
1989 KSS knee score	0.021 (0.979, 1.064)	0.330
1989 KSS function score	0.006 (0.978, 1.035)	0.687
WOMAC pain score	0.054 (1.017, 1.094)	0.004*
WOMAC joint score	0.012 (0.982, 1.043)	0.446
PCS score	−0.006 (0.912, 1.083)	0.889
MCS score	−0.036 (0.878, 1.060)	0.454
2011 symptom score	−0.028 (0.755, 1.252)	0.826
2011 satisfaction score	0.154 (1.080, 1.261)	<0.001*
2011 expectation score	0.539 (1.307, 2.247)	<0.001*
2011 function score	0.067 (1.033, 1.107)	<0.001*

*1989 KSS* 1989 Knee Society Clinical Rating System, *WOMAC* Western Ontario and McMaster Universities Osteoarthritis Index, *SF-12* 12-Item Short-Form Health Survey, *PCS* Physical Component Score, *MCS* Mental Component Score, *2011KSS* 2011 Knee Society Score

*Statistically significant

**Fig. 2:**
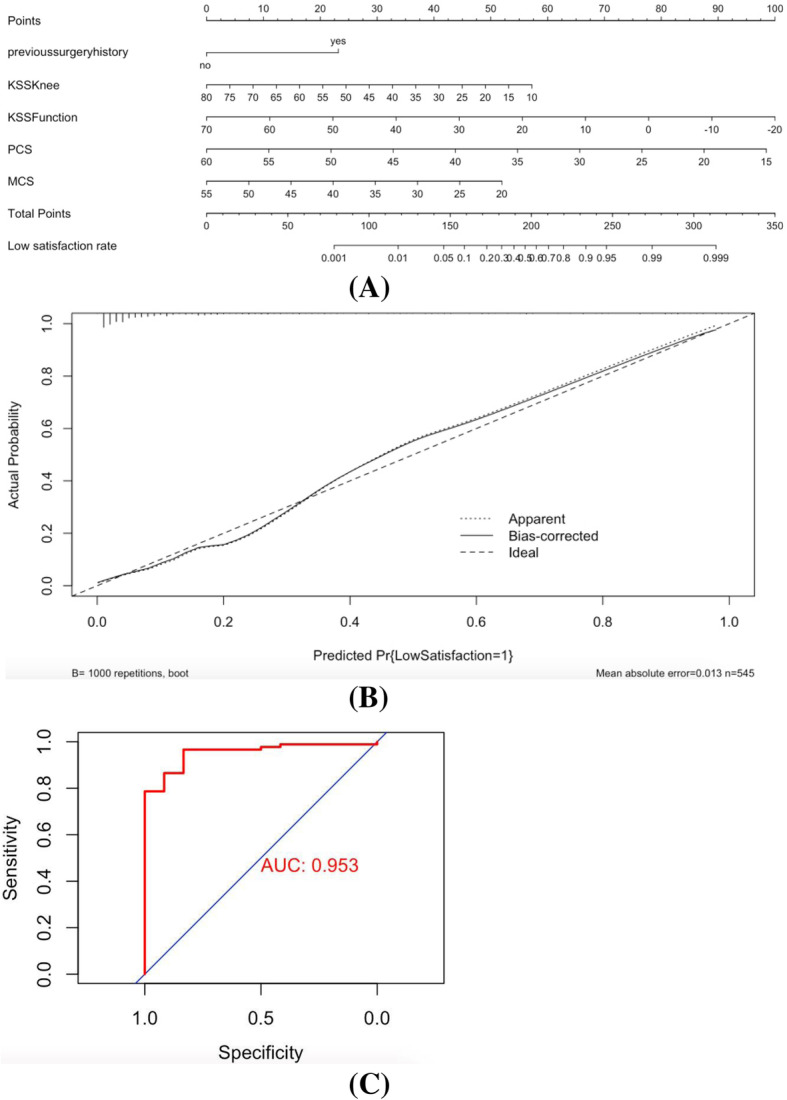
Construction of nomogram. Nomogram for predicting patient satisfaction for the included patients (**a**). Calibration curves of nomograms in terms of agreement between prediction and satisfaction outcomes (**b**). The performance of predictive model in external validation (AUC=0.953) (**c**)

The nomogram demonstrated good accuracy in estimating the risk of low satisfaction after primary TKA, with a C-index of 0.898 > 0.70 (95% confidence interval (CI): 0.86-0.94). The Hosmer-Lemeshow test showed a *p* value of 0.586, suggesting that the model was well fitted (*p* > 0.05). The calibration curve of the nomogram demonstrated good agreement between the predicted and actual satisfaction outcomes in both cohorts after 1000 bootstrap samplings (Fig. 2). In the external validation using the external cohort of 101 patients, the nomogram also displayed good discrimination, with an AUC of 0.953, a sensitivity of 0.87, and a specificity of 0.97 (Fig. 2).

### Roles of different scoring systems in satisfaction

For the postoperative scores and improvement in the scores of scoring systems, linear regression (Table 2) and multivariate logistic regression (Table 3) revealed that some results were correlated to satisfaction level. The potential predictive factors of the four aspects of satisfaction (surgical procedure, functional restoration, pain relief, and fulfillment of expectation) were explored as well (Table 4).

**Table 4:** Predictors of different aspects of patient satisfaction after TKA on multivariate analysis or linear regression analysis

Predictors in different aspects	B (95% CI)	*p* value
Satisfaction with operation		
Preoperative 1989 KSS knee score	0.035 (1.012, 1.059)	0.003
Preoperative 1989 KSS function score	0.036 (1.016, 1.058)	<0.001
Preoperative PCS	0.021 (1.031, 1.120)	0.001
Preoperative MCS	0.021 (1.001, 1.087)	0.046
Change 1989 KSS function score	0.033 (1.015, 1.053)	<0.001
Change WOMAC pain score	0.040 (1.026, 1.056)	<0.001
Change WOMAC function score	0.020 (1.000, 1.041)	0.045
Satisfaction with pain relief		
Change WOMAC pain score	−0.012 (−0.013, −0.010)	<0.001
Satisfaction with functional restoration		
Preoperative 1989 KSS function score	−0.009 (−0.012, −0.007)	<0.001
Change 1989 KSS function score	−0.006 (−0.009, −0.004)	<0.001
Satisfaction of fulfillment of expectation		
2011 KSS expectation score	−0.058 (−0.068, −0.048)	<0.001

*1989 KSS* 1989 Knee Society Clinical Rating System, *WOMAC* Western Ontario and McMaster Universities Osteoarthritis Index, *2011 KSS* 2011 Knee Society Score

### Accuracy and agreement of satisfaction measurements

We compared the assessment of patient satisfaction with the 2011 KSS (the cutoff point of the ROC curve was used to define the satisfaction and dissatisfaction outcomes) with the outcome of overall patient satisfaction by the paired chi-square test and McNemar test. The patient satisfaction obtained from the two methods was dissimilar (*p* = 0.001 < 0.05, 83.7% vs 70.1%), but the agreement between the two methods was general (Kappa=0.437 > 0.4, *p* < 0.001).

## Discussion

We revealed that the prevalence of overall satisfaction postoperatively was ~80%, which is consistent with the data from most reports. We demonstrated that not having undergone surgery before TKA was of utmost importance in achieving patient satisfaction. Compared with other preoperative scoring systems, the 1989 KSS and SF-12 played a vital role in predicting satisfaction. Among the different scoring systems, although most scores and changes in scores could indicate overall patient satisfaction postoperatively, the postoperative outcomes were obviously better tools, and the WOMAC pain subscale score had an explicit advantage among the scoring systems we tested. The predictors of different aspects of patient satisfaction also varied according to the focus of the question on satisfaction.

Traditionally, the outcomes of TKA have been assessed by surgeons using non-validated scoring systems. However, in recent decades, patient-reported outcomes have become popular for assessing postoperative outcomes [2, 5]. Kahlenberg and colleagues showed that most studies use variable methods for measuring and reporting satisfaction, and researchers should focus on standardizing the reporting of patient satisfaction and defining ways to optimize patient satisfaction after TKA [9]. We not only compared the most commonly used method with the validated method of measuring patient satisfaction but also provided suggestions to promote satisfaction levels. This approach reinforces the importance of choosing different scoring systems to predict or reflect patient satisfaction at different periods.

Patient satisfaction 1 year after primary TKA was 83.7%, which is consistent with that reported recently [3, 4, 23, 24]. The predictive factors of low satisfaction after TKA include age, sex, BMI, and expectations. Age and BMI have been controversial factors. Giesinger et al. found a negative impact of BMI on postoperative improvement in satisfaction scores [25]. However, we found that the average BMI of the two groups was very similar (26.01±3.47 vs 26.21±3.58), which indicated that obese or overweight patients might not tend to be dissatisfied after TKA. Usually, patients < 55 years are regarded as young patients in TKA. Lange et al. suggested that the satisfaction rate of young patients (< 55 years) is lower than that of older patients but still higher than 80% [26], while some studies showed that age < 55 years is not an independent predictor of functional recovery or patient satisfaction [27, 28]. In our study, there were only 3 patients < 55 years, so we could not completely judge the difference between young patients and old patients. Female patients accounted for a large proportion in this study (73.9%), but the statistical results showed that sex could not be used as a preoperative predictor, which was consistent with the results of some other studies [2, 3, 29]. Other factors, such as complications and living status, were similar to the overall results, and they could not be used as predictors of satisfaction.

We found that most scoring systems reliably reflected patient satisfaction. There was a correlation between satisfaction and improvements in the pain and physical function outcomes in different scoring systems. For preoperative factors, Graham S et al. showed the reliability of the KSS score in predicting early postoperative satisfaction after TKA [9], and the SF-12 score was an important scoring system for predicting and improving patient satisfaction in most studies [7, 21, 28]. Our study also used these two scoring systems to build a prediction model, and its performance in both the internal and external validation cohorts was excellent. Walker et al. suggested that the WOMAC scoring system could effectively reflect postoperative satisfaction [21], but all subscales in the WOMAC could not be used as predictive factors of satisfaction, which was in accordance with the results of our research.

For postoperative factors, Giesinger et al. showed that the WOMAC pain score and total score were the most important indicators of patient satisfaction 1 year after the operation [30]*.* Several reports have ignored the influence of the pain score in the WOMAC, but they used too few scoring systems to discover significant differences [7, 31]. In our study, the WOMAC pain score was the only subscale that showed differences in both the change scores and postoperative scores. We recommended it to reflect overall patient satisfaction 1 year after TKA.

The predictors of different major aspects of patient satisfaction after primary TKA varied because of the lack of a clear definition of dimensions surgeons should pay attention to. Mahomed et al. developed and validated a method for evaluating overall satisfaction, as well as satisfaction with pain relief, the ability to do housework, and with the ability to undertake recreational activities. However, they did not include the fulfillment of expectation, which has been shown to be vital for patient satisfaction [32]. We discovered the potential predictive factors of different focuses of satisfaction, but they still need further validation.

The primary strength of our study was a general comparison of different scoring systems preoperatively and postoperatively, which highlights the importance of the 1989 KSS and SF-12 scores preoperatively and the WOMAC scores postoperatively. Based on our findings, surgeons can adjust their patient management strategy before and after surgery to achieve a higher prevalence of patient satisfaction after primary TKA. In addition, our study suggests the possibility of predicting patient satisfaction through previous surgical history. More specific predictive factors might be related to the follow-up time, the time of previous operations, and even the satisfaction level of previous operations, so further research is needed. In addition, for the first time, we compared the following methods of measuring satisfaction: patient satisfaction from the 2011 KSS [33, 34] and a single question of overall satisfaction.

Our study had five main limitations. First, the sample size was small, and only 75.5% of eligible patients completed all required forms. Second, our data may be unrepresentative of the general population and raise the risk of biases. Third, we assessed patient expectations only by asking whether expectations had been met or not, without the evaluation of expectations preoperatively. Fourth, we only compared different scoring systems to discover the possible predictors of various aspects of patient satisfaction; we did not analyze demographic or clinical data. Fifth, we assessed patient satisfaction 1 year after TKA. The perception and feeling of pain and function may continue to improve and promote the level of satisfaction, so a longer follow-up would have been a good strategy.

## Conclusions

A history of surgery, the preoperative 1989 KSS, and the preoperative SF-12 influenced patient satisfaction after primary TKA. We recommend the WOMAC (particularly the pain subscale score) to reflect overall patient satisfaction postoperatively.

## Data Availability

The datasets generated and/or analyzed during the current study are not publicly available due to the data application regulations of the hospital but are available from the corresponding author on reasonable request.
